# Lymphangiography and focal pleurodesis treatment of chylothorax with an aberrant thoracic duct following oesophagectomy: a case report

**DOI:** 10.1186/s40792-019-0709-3

**Published:** 2019-12-11

**Authors:** Tomoyuki Ishida, Jun Kanamori, Hiroyuki Daiko

**Affiliations:** Department of Esophageal Surgery, National Cancer Center Hospital, 5-1-1 Tsukiji, Chuo-ku, Tokyo, 104-0045 Japan

**Keywords:** Thoracic duct, Chylothorax, Oesophageal cancer, Variation

## Abstract

**Background:**

Management of postoperative chylothorax usually consists of nutritional regimens, pharmacological therapies such as octreotide, and surgical therapies such as ligation of thoracic duct, but a clear consensus is yet to be reached. Further, the variation of the thoracic duct makes chylothorax difficult to treat. This report describes a rare case of chylothorax with an aberrant thoracic duct that was successfully treated using focal pleurodesis through interventional radiology (IVR).

**Case presentation:**

The patient was a 52-year-old man with chylothorax after a thoracoscopic oesophagectomy for oesophageal cancer. With conventional therapy, such as thoracostomy tube, octreotide or fibrogammin, a decrease in the amount of chyle was not achieved. Therefore, we performed lymphangiography and pleurodesis through IVR. The patient appeared to have an aberrant thoracic duct, as revealed by magnetic resonance imaging (MRI); however, after focal pleurodesis, the leak of chyle was diminished, and the patient was discharged 66 days after admission.

**Conclusions:**

Chylothorax remains a difficult complication. Focal pleurodesis through IVR can be one of the options to treat chylothorax.

## Background

Postoperative chylothorax after oesophagectomy occurs relatively infrequently, in approximately 2–9% of patients [1–3]. Management of postoperative chylothorax generally involves nutritional regimens as well as pharmacological and surgical therapies, but a clear consensus has yet to be reached [4].

## Case presentation

**Fig. 1:**
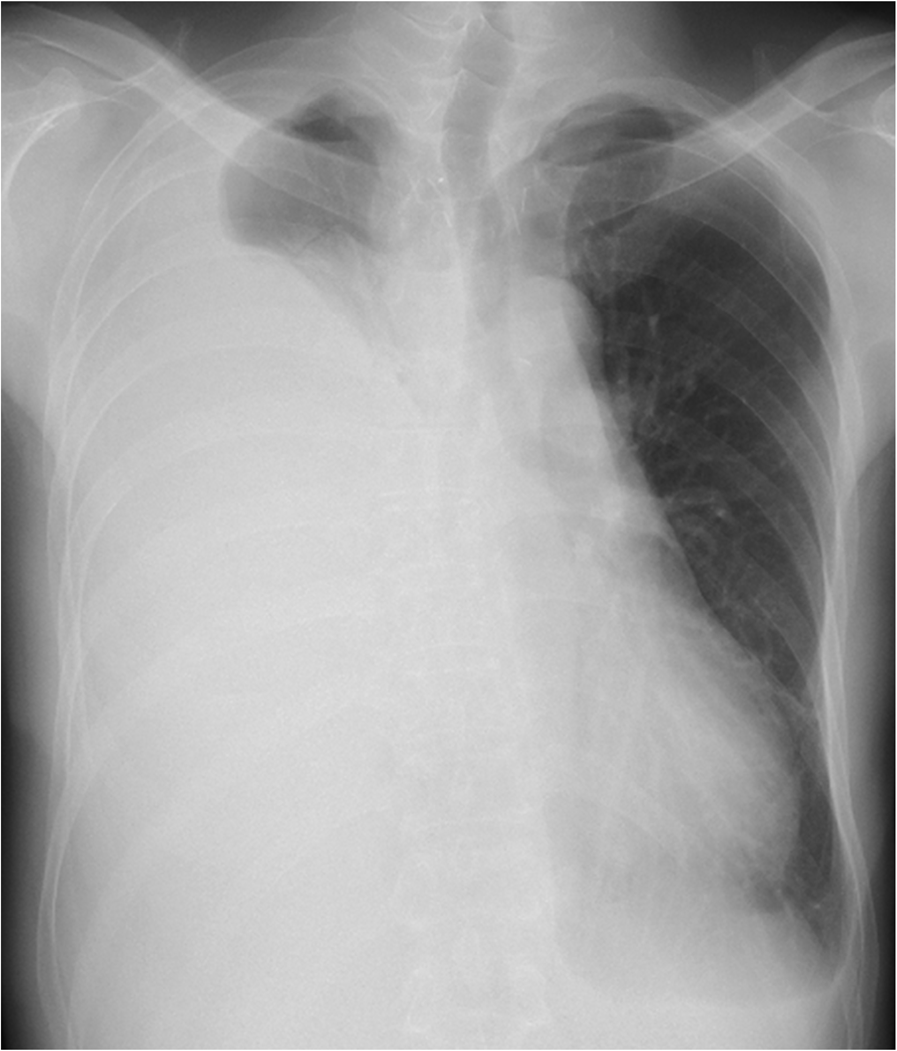
Chest X-ray 1 month after operation revealed large amounts of right-sided pleural effusion

In November of 2017, a 52-year-old patient underwent thoracoscopic oesophagectomy and laparoscopic retrosternal gastric tube reconstruction with lymph node dissection for oesophageal cancer after neo-adjuvant therapy. The thoracic duct was clipped at the level above the diaphragm using a clip applier. The histopathological diagnosis was that there was no residual tumour seen after chemotherapy, and there was no metastatic tumour within dissected lymph nodes (therapeutic effect grade 3). At 1 month of follow-up as an outpatient, chest X-ray revealed a right-sided pleural effusion (Fig. 1). We inserted a thoracostomy tube into the right chest cavity through the intercostal space, and the patient was diagnosed as having chylothorax on the basis of 1000 ml chyle being obtained.

**Fig. 2:**
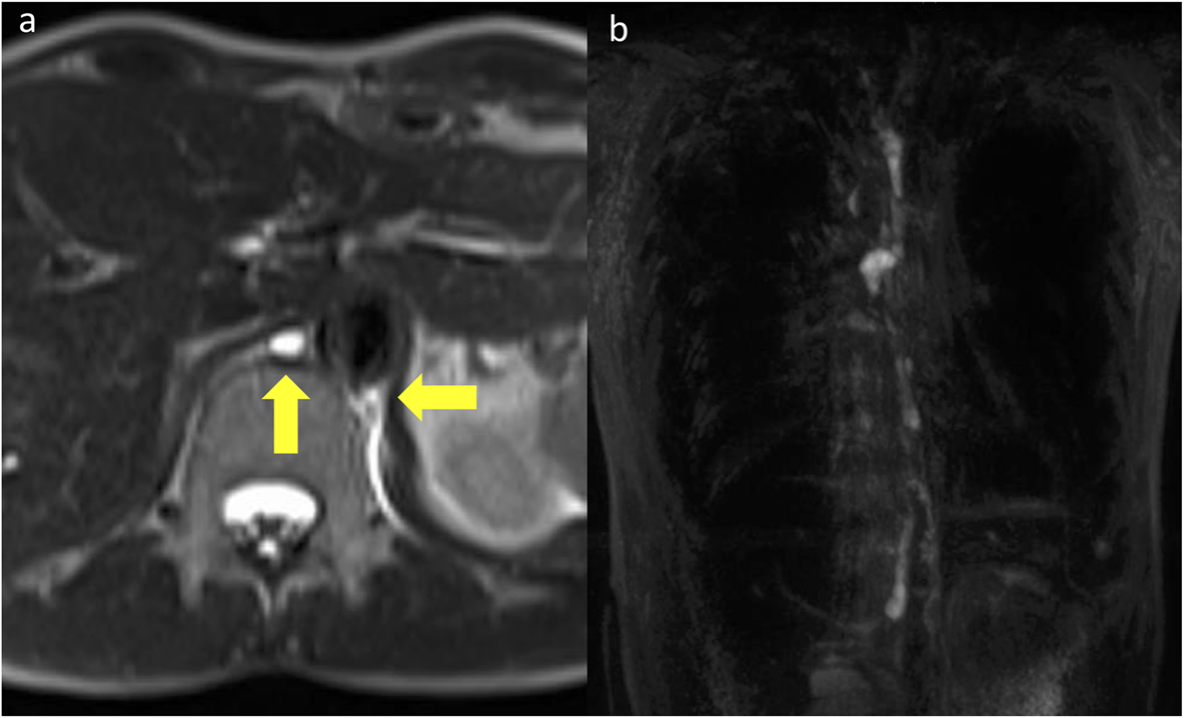
**a** MRI 10 days after admission revealed two thoracic ducts running through the thoracic cavity. **b** Coronal imaging of MRI

We conducted continuous drainage, used octreotide and started total parenteral nutrition. Beginning at 5 days after admission, we use fibrogammin for 3 days, but its effectiveness was insufficient. Magnetic resonance imaging (MRI), taken on the 10th day after admission, detected two thoracic ducts; the right one ended in the thoracic cavity and was thought to be dissected, possibly at the time of operation (Fig. 2). The left one running through behind the aorta is not usually detected, and thus, our case appeared to have an aberrant thoracic duct.

**Fig. 3:**
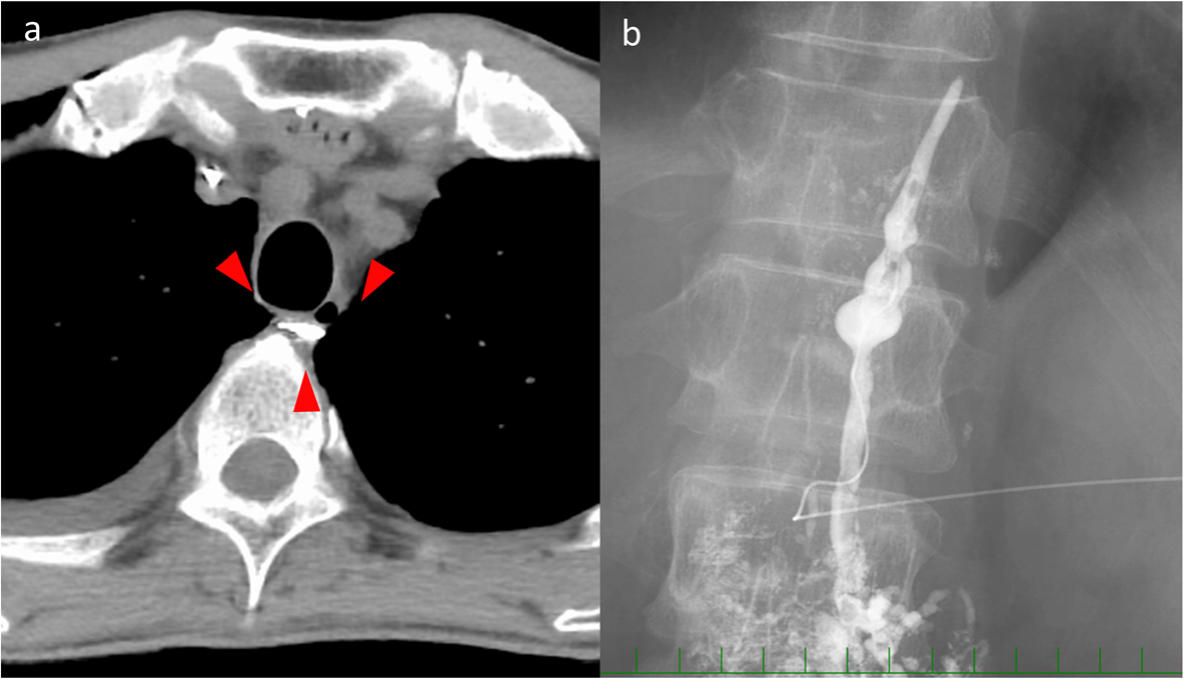
**a** CT image taken after lymphangiography. Contrast agent leaked from thoracic duct at the level of bifurcation of trachea. **b** Lymphangiography through the cisterna chyli revealed thoracic duct was dissected in the thoracic cavity

**Fig. 4:**
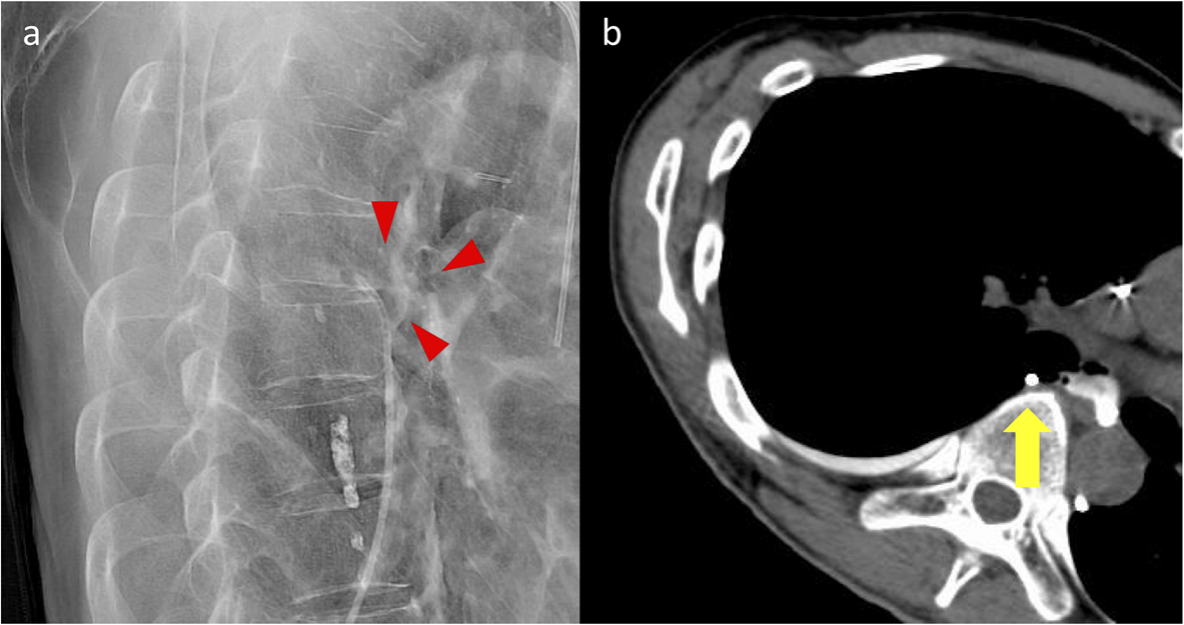
**a**, **b** Pleurodesis with OK-432 through interventional radiology (IVR) from the leakage point

Without improvement of chyle leak, on the 14th day after admission, we conducted lymphangiography through the inguinal region. However, contrast agent did not rise over at the level of L1. On the computed tomography (CT) taken after that procedure, contrast agent leaked from thoracic duct at the level of the bifurcation of the trachea to the right thoracic cavity (Fig. 3a). Next, we conducted lymphangiography through the cisterna chyli. After injecting the contrast agent into the inguinal lymph nodes, the cisterna chyli was drawn gradually. With CT scan, we punctured the cisterna chyli with a 21-gauze needle. The thoracic duct was dissected in the thoracic cavity, and extravasation was not observed (Fig. 3b). However, the amount of pleural effusion was gradually decreased, and we finished the octreotide on the 25th day after admission and started a fat-restricted diet the next day. Because the amount of drainage fluid increased again on the 29th day after admission, we stopped the meals again. Another lymphangiography was conducted, but it was not sufficiently effective. Though we punctured the cisterna chyli again, expected embolization of the thoracic duct was not achieved. Therefore, we transported the narrow tube to the leakage point through interventional radiology (IVR) and performed pleurodesis with OK-432 on the 45th and 48th days after admission (Fig. 4). After the clinical adverse effects like fever around 38 degrees and back pain in a few days, the amount of chyle leakage decreased. Next, we started the fat-restricted diet on the 55th day after admission, and the next day, we started normal meals. The thoracic drain was removed on the 62nd day after admission. On the 66th day after admission, he was discharged from the hospital.

## Discussion

Chylothorax is one of the complications after oesophagectomy, but remains a rare complication. The frequency of chylothorax is reported to be between 1.1–2.7% after oesophageal resection [5, 6].

Chylothorax treatment after oesophagectomy is troublesome itself, and with an aberrant thoracic duct, the treatment becomes more difficult. In 1953, Adachi reported classification of thoracic duct variations with 9 types of normal anatomy [7]. In our case, there were right and left thoracic ducts; the right one was clipped in the operation of oesophageal cancer, but the left one remained. Thus, we classified this case to the type III Adachi thoracic duct classification.

Regarding whether to resect the thoracic ducts or preserve them in oesophageal resection, there have been few reports on the efficacy of thoracic duct resection [8], and a consensus of resection of thoracic duct has yet to be reached. In our hospital, we usually clip and dissect the thoracic duct at the level above the diaphragm for oesophageal cancer; however, postoperative chylothorax still remains a rare complication.

Conservative therapy is initially suggested. The treatment consists of thoracic cavity drainage, nutritional support, pleurodesis, and measures to diminish chyle flow. If conservative treatment is not successful, in the following cases, surgical treatment is chosen: chyle leak continuing, nutritional status deteriorating and the possibility of infection increasing. In adult cases, Selle et al. [9] reported the standard that in cases with over 1500 ml fluid flowing out over 5 days, conservative treatment over 2 weeks and nutritional status deterioration are adaptations of surgical treatment. We conducted lymphangiography and focal pleurodesis because conservative therapy continued over 2 weeks. In previous studies, conservative treatment (excluding thoracic duct embolization) had a success rate of 53.8% in the postoperative chylothorax [3, 10–15], with or without thoracic duct resection.

We watched and investigated the operation video carefully after the appearance of chylothorax; the thoracic duct was clipped and dissected conventionally at that time. Thus, we would consider that there was an aberrant thoracic duct, which is not usually the case. In the case of minimal invasive oesophageal resection, it is very useful to re-examine the operation video precisely after incidence of complications.

The thoracic duct is the largest lymphatic vessel in the human body [16]. Because of its proximity to other organs, such as the oesophagus, the thoracic duct is at risk during surgery. Routine ligation of the thoracic duct is advocated to prevent chyle leakage [17, 18]. However, thoracic chyle leakage still occurs, even when the thoracic duct is clipped [19]. There are many variations in thoracic duct anatomy. In our case, MRI showed thoracic duct variation. Above the diaphragm, the normal thoracic duct runs along the right side of the thoracic vertebrae and the dorsal side of oesophagus, and between the thoracic aorta and azygos vein. Around the 6th to 4th thoracic vertebrae, it passes behind the oesophagus through the thoracic vertebrae and enters into left posterior mediastinum. Finally, it goes through upper mediastinum to neck, and then, it goes down and enters into the left vein angle [20]. In the lymphangiography, Cha and Sirijintakarn [21] reported that the frequency of variation is 26.8% (65 cases of 243 cases), and Asada et al. [22] reported a frequency of variation of 29% (60 cases of 207 cases). When chylothorax occurs, lymphangiography is needed because of the possibility of variation, detecting the thoracic duct run and leaking point [23, 24].

In this case, we transported the narrow tube to the leakage point and have shown the effectiveness of focal pleurodesis through IVR, in combination with conventional octreotide administration and nutritional therapy, for the treatment of postoperative chylothorax following oesophagectomy. Our finding suggests that when used concurrently with conventional treatments, focal pleurodesis facilitates early chest tube removal and there is no need of surgical treatment with or without thoracic duct variation.

## Conclusions

Focal pleurodesis through IVR can be one of the options to treat chylothorax, with or without thoracic duct variation. When the chyle is not diminished after several conventional treatments, surgeons should be mindful of the possibility of focal pleurodesis.

## Data Availability

No applicable.

## Abbreviations

CT: Computed tomography
IVR: Interventional radiology
MRI: Magnetic resonance imaging
